# The *SEEDLING BIOMASS 1* allele from *indica* rice enhances yield performance under low‐nitrogen environments

**DOI:** 10.1111/pbi.13642

**Published:** 2021-06-13

**Authors:** Jing Xu, Lianguang Shang, Jiajia Wang, Minmin Chen, Xue Fu, Huiying He, Zian Wang, Dali Zeng, Li Zhu, Jiang Hu, Chao Zhang, Guang Chen, Zhenyu Gao, Weiwei Zou, Deyong Ren, Guojun Dong, Lan Shen, Qiang Zhang, Qing Li, Longbiao Guo, Qian Qian, Guangheng Zhang

**Affiliations:** ^1^ State Key Laboratory of Rice Biology China National Rice Research Institute Hangzhou China; ^2^ Lingnan Laboratory of Modern Agriculture Genome Analysis Laboratory of the Ministry of Agriculture Agricultural Genomics Institute at Shenzhen Chinese Academy of Agricultural Sciences Shenzhen China

**Keywords:** rice, biomass, grain number, nitrogen utilization efficiency, grain yield

Since the ‘first green revolution’ in the 1960s, rice grain yield has risen sharply. However, due to the continual decreasing of cropping areas, further increase in yield potentials is urgently demanding. Long‐term excessive fertilization has led to uncontrolled retention of nitrogen fertilizer in the soil and serious pollution of the environment (Peng *et al*., 2002). Thus, identification of genes determining both high yield and improved nitrogen utilization efficiency for genetic modification is a necessary and promising approach to breed new desirable varieties suitable for current rice production. In this study, we aimed at a primary‐mapping QTL controlling rice seedling biomass on chromosome 1 (*qSBM1*) detected in the recombinant inbred lines derived from a cross between 93‐11 and PA64S and cloned the underlying gene (*LOC_Os01g65120*) by using near‐isogenic lines (NILs) containing Kasalath allele at *qSBM1* (NIL‐*qSBM1* ^Kasalath^) in the Nipponbare (NPB) background as well as a series of transgenic lines (Figure 1a, b). *SBM1* was shown to significantly affect many yield‐related traits besides the seedling biomass.

**Figure 1:**
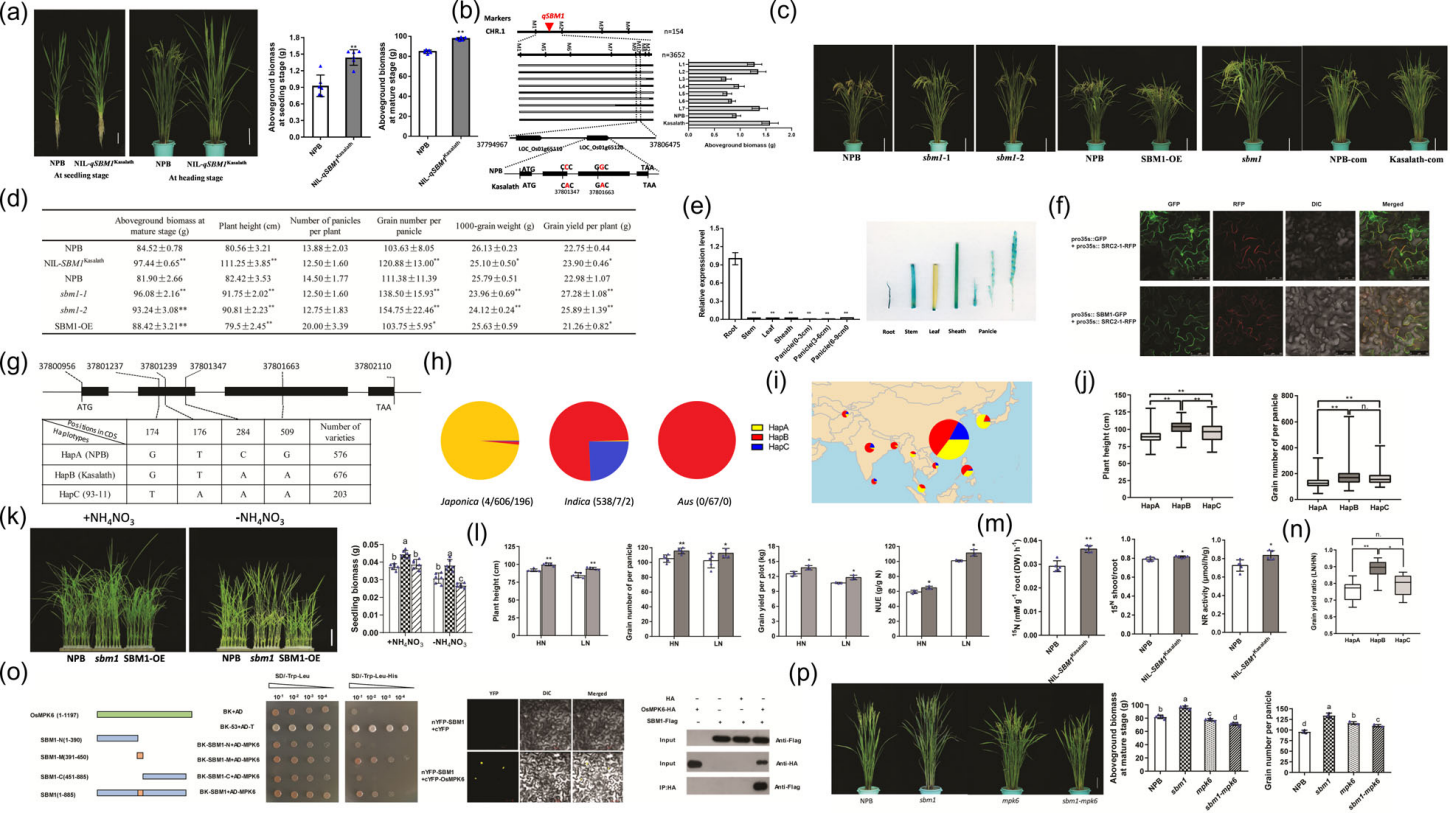
*SBM1* controls yield traits in rice. (a) Phenotype of NIL‐*qSBM1* ^Kasalath^ and its recurrent parent Nipponbare (NPB) at seedling and heading stages. Bar = 10 cm. (b) Map‐based cloning *SBM1*. (c) Transgenic test by CRISPR/Cas9, overexpression (OE) and complementation (com). (d) Statistical analysis of important agronomic traits in NPB, NIL‐*SBM1* ^Kasalath^, knock‐out mutants (*sbm1*), SBM1‐OE lines. (e) Expression pattern of *SBM1* via qRT‐PCR and *GUS* staining. (f) Subcellular localization of SBM1 in leaf epidermal cells of *N.benthamiana*. Bar = 50 μm. (g) Three haplotypes (HapA‐C) of single nucleotide polymorphisms (SNPs) in the *SBM1* coding region. (h) Distribution of HapA‐C in rice subgroups of *japonica*, *indica* and *aus*. HapA, HapB and HapC are coloured by yellow, red and blue, respectively. The number of accessions in each haplotype is shown in brackets for each rice subgroup. (i) Geographic distributions of HapA‐C. (j) Plant height and grain number of per panicle among HapA, HapB and HapC. (k) Phenotype of NPB, *sbm1*, *SBM1*‐OE plants grown in presence (left) or absence (right) of NH_4_NO_3_ nutrient solution for 20 days. Bar = 5 cm. (l) Important agronomic traits analysis between NPB and NIL‐*SBM1* ^Kasalath^ grown under nitrogen‐limiting growth conditions. (m) Comparison of nitrogen uptake using ^15^N‐NH_4_NO_3_, ^15^N root‐to‐shoot transport and NR activity between NPB and NIL‐ *SBM1* ^Kasalath^. (n) Grain yield ratio among accessions of HapA, HapB and HapC grown under low‐nitrogen to high‐nitrogen conditions. (o) Interaction analysis between SBM1 and OsMPK6 detected by yeast two‐hybrid assays, bimolecular fluorescent complimentary (BiFC) and co‐immunoprecipitation. (p) Phenotype and aboveground biomass and grain number per panicle of knock‐out mutants (*sbm1*), *mpk6* and double mutant (*sbm1‐mpk6*).

Compared to NPB, the NIL‐*qSBM1* ^Kasalath^ showed significant increase in biomass aboveground, plant height, grain number per panicle and grain yield per plant, but significant decrease in 1000‐grain weight, with no significant difference in panicle number per plant (Figure 1d). Similar but much greater trait variation tendency in knock‐out (ko) mutants of *SBM1* (*sbm1*) generated by using CRISPR/Cas9 technology. As expected, the overexpression (OE) transgenic SBM1‐OE plants showed opposite trait variation tendency to the NIL‐*qSBM1* ^Kasalath^ and *sbm1* when compared to NPB (Figure 1c, d). Additionally, to ensure the phenotype of ko mutants was caused by the mutation of *SBM1*, complementation constructs (com) which harboured the promoter and coding sequence of *SBM1* from either NPB or Kasalath were generated and introduced into ko lines. We found that the NPB‐com transgenic plants rescued the phenotype of ko mutants, while the Kasalath‐com showed a partially rescued phenotype which was similar to NIL‐*qSBM1* ^Kasalath^ (Figure 1c). qPCR and *GUS* staining showed that *SBM1* was expressed in root, stem, leaf, sheath and panicle at different development stages, with preferential expression in roots (Figure 1e). SBM1‐GFP was localized at plasma membrane and co‐localized with the plasma membrane localization marker PM‐SRC2‐1 (Liu *et al*., 2015a) (Figure 1f).

Three main haplotypes (Haps) of *SBM1*, such as HapA (NPB, GTCG), HapB (Kasalath/PA64s, GTAA) and HapC (93‐11, TAAA), were identified in 1140 rice accessions of broad genetic diversity from Xie *et al*. (2015) (Figure 1g), which could be divided into two major subspecies, *japonica* and *indica*. HapA was widely distributed in *japonica* rice, while HapB and HapC were mainly in *indica* rice, and notably, *aus* rice, a subpopulation of *indica*, was majorly comprised of rice accessions with HapB (Figure 1h). The geographic pie chart suggested that the haplotype might be originated from Bangladesh (Figure 1i). Then, based on the plant height, and grain number per panicle of rice accessions we collected with the three haplotypes, HapB was shown to be the most productive (Figure 1j). These results further confirmed the role of the four causative SNPs played in phenotypic variations.

Given that *SBM1* encodes a plasma membrane‐localized oligopeptide transporter domain containing protein (Figure 1f), it may be involved in nitrogen utilization (Hu *et al*., 2015). We selected NPB, ko and OE plants to test the sensitivity of *SBM1* to nitrogen treatment. Regardless of the presence of NH_4_NO_3_ or not, compared to NPB, both ko and OE lines showed significant difference in seedling biomass, with increase and decrease, respectively (Figure 1k). These indicated that *SBM1* might respond to varied nitrogen application. Next, we tested this inference through a field experiment by using NPB and NIL‐*SBM1^Kasalath^*, with two different nitrogen application rates, *that is* high‐nitrogen (180kg/ha urea) and low‐nitrogen (90kg/ha urea) conditions. Compared to NPB, NIL‐*SBM1^Kasalath^* showed significant increase in plant height, grain number per panicle and grain yield per plot, but not in tiller number per plant under both conditions (Figure 1l). Given that significantly higher NUE was observed in NIL‐*SBM1^Kasalath^* (Figure 1l), we tried to test the difference in nitrogen uptake and transport activity between NPB and NIL‐*SBM1^Kasalath^* through a ^15^N‐NH_4_NO_3_ feeding experiment. NIL‐*SBM1^Kasalath^* showed significantly higher ^15^N uptake and transport activity than NPB (Figure 1m). Also, nitrate reductase activity, an important indicator of nitrogen utilization, was higher in NIL‐*SBM1^Kasalath^* than in NPB (Figure 1m). These confirmed the sensitivity of *SBM1* to varied nitrogen application. Additionally, through phenotyping, rice accessions with Kasalath haplotype (HapB) at *SBM1* tended to have a higher grain yield ratio (LN/HN) (Figure 1n), indicating this haplotype had great potential for improving nitrogen utilization in rice, as shown by desirable yield performance under low‐nitrogen conditions.

To further reveal the genetic pathway *SBM1* mediated, we used yeast two‐hybrid screening to identify candidate interacting genes. OsMPK6 that regulated biomass and grain traits ( Liu *et al*., 2015b) was shown to interact with SBM1, which mainly occurred in the middle region of 391–450 bp in the *SBM1* cDNA (Figure 1o). Then, their interaction was supported by bimolecular fluorescence complementation (BiFC) and co‐immunoprecipitation (Co‐IP) (Figure 1o). To confirm the genetic relationship between *SBM1* and *OsMPK6*, the *ko* mutants of the two genes were obtained by CRISPR‐Cas9 technology in the NPB background, including the single mutant *sbm1* and *mpk6* and the double mutant *sbm1‐mpk6* (Figure 1p). Compared to NPB, significant larger and smaller biomass were found in *sbm1* and *mpk6*, respectively, while significant more grains per panicle were found in both mutants (Figure 1p). Notably, in the double mutant *sbm1‐mpk6*, the negative role of *SBM1* in biomass formation was not observed, and the greatly increasing effect of the *SBM1* null mutation on grain number was also weakened. These indicated the larger biomass and more grains in the *sbm1* mutant genetically depended on *OsMPK6*. Given that *OsMPK6* was also responsible for resistance to abiotic and biotic stresses (Ma *et al*., 2021) and its homologous gene *MPK6* in *Arabidopsis* could modulate nitrate reductase activity (Wang *et al*., 2011), the significance of the *SBM1‐OsMPK6* mediated pathway was highlighted, and the function of *SBM1* on yield performance and nitrogen utilization described above were further confirmed.

In summary, the application of *SBM1*, a pleiotropic gene responsible for yield traits, plant size and nitrogen utilization efficiency, could greatly contribute to breeding new high‐yield rice varieties, as *NGR5, OsNR2*, *OsNRT1.1B* and *GRF4* did (Gao *et al*., 2013; Hu *et al*., 2015; Li *et al*., 2018; Wu *et al*., 2020). Notably, the four genes enhanced grain yield mainly through increasing panicle number per plant, while *SBM1* improved yield performance by increasing grain number per panicle. Meanwhile, given its another characteristic of enhancing nitrogen utilization, *SBM1* would improve yield performance at low‐nitrogen supply, especially if introgressing the HapB of *SBM1* to *japonica* rice that lacks this favourable haplotype. Additionally, the interaction between *SBM1* and *OsMPK6* provides new leads to explore the underlying molecular mechanism.

## Conflict of interest

The authors declare no conflict of interest.

## Author contributions

Qian Qian and Guangheng Zhang designed this research; Jing Xu, Lianguang Shang, Jiajia Wang, Minmin Chen, Xue Fu, Huiying He, Zian Wang, Dali Zeng, Li Zhu, Jiang Hu, Chao Zhang, Guang Chen, Zhenyu Gao, Weiwei Zou, Deyong Ren, Guojun Dong, Lan Shen, Qiang Zhang, Qing Li and Longbiao Guo performed the experiments; Jing Xu, Lianguang Shang and Guangheng Zhang wrote the manuscript.
